# Unruptured Ectopic Pregnancy at Eight Weeks in a Woman With an Intrauterine Device

**DOI:** 10.7759/cureus.109535

**Published:** 2026-05-24

**Authors:** Luísa Cunha Silva, Maria Rui Torres, Joana Araújo Pereira, João Pedro Prata, Paula Pinheiro

**Affiliations:** 1 Obstetrics and Gynaecology, Unidade Local de Saúde do Alto Minho, Viana do Castelo, PRT

**Keywords:** contraception, fallopian tubes, intrauterine contraceptive device (iud), tubal ectopic pregnancy, unruptured ectopic pregnancy

## Abstract

Ectopic pregnancy, a rare entity, represents a significant cause of maternal morbidity and mortality in the first trimester, with the fallopian tube being the most common implantation site. While intrauterine devices (IUDs) are highly effective contraceptives that reduce overall pregnancy risk, contraceptive failure with an IUD in situ is associated with a slightly higher rate of ectopic pregnancy compared to women not using contraception.

This case report describes an 8-week and 4-day unruptured tubal ectopic pregnancy in a 38-year-old multiparous woman with a well-positioned 52-mg levonorgestrel-releasing intrauterine device (L-IUD) that had been in place for one year.

The patient presented with breast tenderness and a positive pregnancy test but was otherwise asymptomatic, with no pelvic pain or vaginal bleeding. She underwent successful laparoscopic bilateral salpingectomy and was discharged within 24 hours without complications.

This case highlights the importance of maintaining clinical vigilance for ectopic pregnancy in women with IUDs, even in the absence of typical symptoms, and demonstrates that advanced gestational age tubal pregnancies can remain unruptured. Clinicians should consider ectopic pregnancy from the differential diagnosis in asymptomatic women with IUDs who present with a positive pregnancy test.

## Introduction

Ectopic pregnancy (EP) is a pregnancy in which the developing blastocyst becomes implanted at a site other than the endometrium of the uterine cavity [1]. Ninety-six percent of EPs occur in the fallopian tube [1]. Ectopic pregnancy is an important cause of women’s mortality during the first trimester of pregnancy [2].

The major cause of EP is the disruption of the normal tubal anatomy. This disruption can result from many factors, such as infection, infertility, congenital anomalies, or a history of prior ectopic pregnancy or tubal surgery [1]. Patients using hormonal contraception or an intrauterine device (IUD) are at very low risk of conceiving a pregnancy, either intrauterine or ectopic [1,3]. However, if they conceive a pregnancy, the probability of an EP is generally higher than in those not using contraception [1]. This may be explained by changes in the motility of the Fallopian tubes, caused by the progestative effect, as well as mechanical changes that the levonorgestrel-releasing IUD (L-IUD) causes in the uterine cavity, not favoring uterine implantation [3].

Schultheis et al. report that the risk of EP in women bearing an L-IUD (52 mg L-IUD) is 0.5 per 1000 women-years, compared with 6.9 in women using no contraception or barrier methods. Rates were similarly low with the copper IUD (0.46), oral contraceptives/contraceptive patch/vaginal ring (0.22), and contraceptive implant/depot medroxyprogesterone acetate (0) [3]. The literature indicates that the 52 mg L-IUD has a lower risk of EP than the 13.45 mg L-IUD [4].

Ectopic pregnancy tubal location increases the risk of tubal rupture to 29%, according to the literature [1,2].

## Case presentation

The patient was a 38-year-old woman with a history of ulcerative colitis treated with mesalamine, no prior surgical history, and no known drug allergies. She was a multiparous woman with a history of two full-term pregnancies and two normal deliveries. She was a user of a 52 mg L-IUD, placed one year before, with no complications reported and amenorrhea since placement.

She presented to the Gynecology and Obstetrics emergency department due to breast tenderness. No pelvic or vaginal bleeding was reported. The patient performed a urine pregnancy test, which was positive.

On physical examination, the patient was hemodynamically stable, afebrile, and well perfused. The gynecological examination was unremarkable, with visible L-IUD strings on speculum examination.

**Figure 1:**
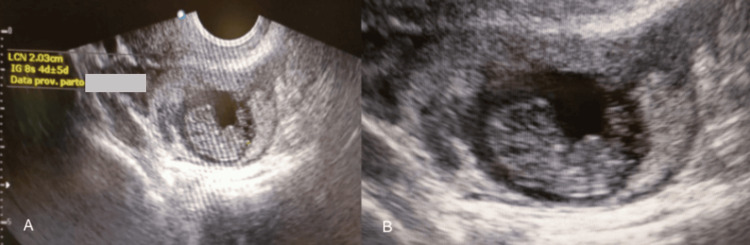
Transvaginal ultrasound Transvaginal ultrasound performed on admission, showing right tubal ectopic pregnancy, embryo with crown-rump length of 20 mm, compatible with 8 weeks and 4 days and positive embryocardium.

A pelvic ultrasound was performed and showed an anteverted uterus without endometrial thickening and an L-IUD in situ within the uterine cavity. Adnexal evaluation revealed no abnormalities in the left adnexa and a normal right ovary; in the right tube, a regular gestational sac was visualized with an embryo with a crown-rump length (CRL) of 20 mm compatible with 8 weeks and 4 days of gestation, with positive embryocardium (Figure 1). In the pouch of Douglas, a small amount of free fluid was noted.

**Figure 2:**
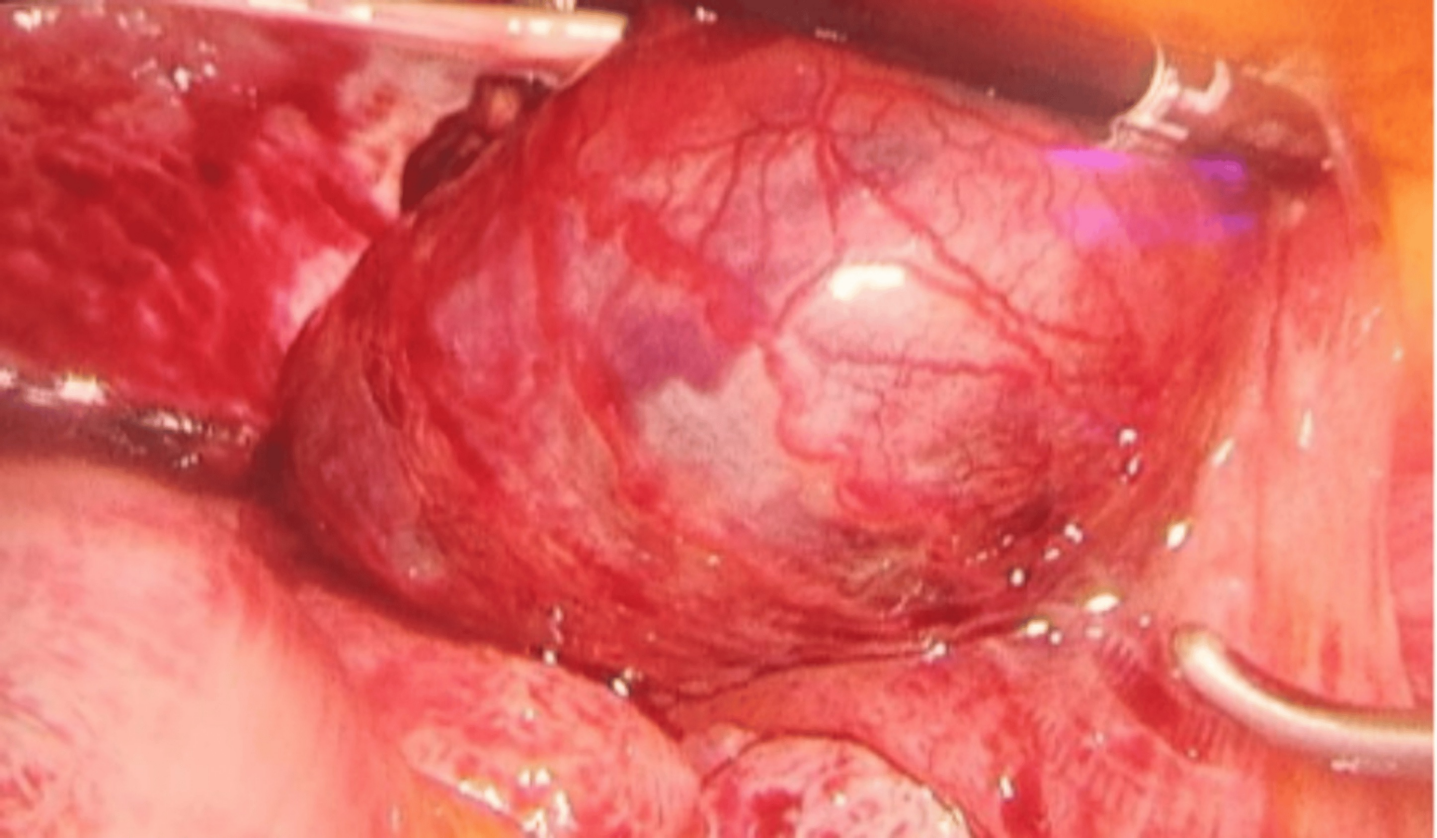
Laparoscopic findings Image of the pelvic cavity, obtained by laparoscopic surgery, demonstrating a swollen and vascularized right fallopian tube, without rupture.

Laparoscopic surgical treatment of the unruptured tubal ectopic pregnancy was performed (Figure 2).

Because the patient desired sterilization, a bilateral total salpingectomy was performed rather than a unilateral procedure (salpingectomy/salpingostomy). The patient was discharged 24 hours after surgery, asymptomatic, and the L-IUD was maintained to preserve amenorrhea.

At the six-week follow-up, the patient remained asymptomatic and amenorrheic.

## Discussion

In the case presented, a tubal EP is described, located in the most frequent site of extrauterine pregnancies [1]. The conception occurred in a woman with an L-IUD in situ, even though the probability of pregnancy in a woman with an L-IUD is low (0.2% in the first year)[5]. After method failure, the probability of ectopic pregnancy increases in relation to women who become pregnant without contraception [3,5].

With tubal EP, the probability of tubal rupture is approximately 29%, and the risk increases as the gestational age increases [1,2]. The most common time for rupture of a tubal ectopic gestation is between six and nine weeks of gestation, although this can vary depending on the site and size of implantation and clinical factors [6]. Rupture can result in life-threatening intra-abdominal hemorrhage with severe or persistent abdominal pain and/or hemodynamic instability [7].

In the presented clinical case, we highlight the gestational age, 8 weeks and 4 days, and the absence of tubal rupture, as well as the total absence of the most common signs and symptoms associated with this entity, vaginal bleeding and/or abdominal pain.

The choice of treatment in tubal ectopic pregnancy depends on several factors, including hemodynamic stability, serum beta-human chorionic gonadotropin levels, gestational age, sonographic findings, the presence or absence of fetal cardiac activity, tubal status, and the patient’s reproductive wishes. Management options include expectant management, medical treatment with methotrexate, and surgical intervention - salpingectomy or salpingostomy. Hemodynamically unstable patients or those with evidence of tubal rupture require immediate surgical treatment, whereas clinically stable patients may be candidates for medical management if the ectopic mass is small, there is no fetal cardiac activity, and no sonographic evidence of rupture is present. Expectant management may be considered only in carefully selected stable patients with declining serum beta-human chorionic gonadotropin levels and minimal symptoms under close follow-up [8].

In the present case, a laparoscopic bilateral salpingectomy was performed due to the presence of embryonic cardiac activity and the patient’s desire for definitive sterilization.

In the literature, the number of reported cases of ectopic tubal pregnancy decreases as gestational age increases, with published cases, including a twin pregnancy at 8 weeks and 4 days and a separate pregnancy at 14 weeks [9]. In other extra-uterine locations, there are reports of pregnancies with a much higher gestational age.

To the authors' knowledge, there are few case reports associating the presence of an L-IUD in situ with a tubal EP with a gestational age similar to or greater than the present case [2].

## Conclusions

In all women of reproductive age, pregnancy should be considered and excluded early in the assessment process, regardless of reported contraceptive use. This case highlights that, despite high contraceptive efficacy, practitioners must maintain a high index of suspicion for ectopic implantation in any IUD user with a positive pregnancy test. It also illustrates that tubal pregnancies can progress to advanced gestational ages without rupture or significant symptoms, emphasizing the need for suspicion, a thorough gynecological assessment, and early ultrasound evaluation.

Ultimately, comprehensive patient counseling is essential. Women choosing IUDs should be informed about the efficacy of these devices but also about their complications in the short, middle, and long term, including the risk of ectopic pregnancy and the need to seek prompt medical evaluation if pregnancy is suspected. Early recognition and diagnosis of ectopic pregnancy in IUD users can prevent life-threatening complications and allow for timely, appropriate management.
